# Physical Activity during Work, Transport and Leisure in Germany - Prevalence and Socio-Demographic Correlates

**DOI:** 10.1371/journal.pone.0112333

**Published:** 2014-11-12

**Authors:** Birgit Wallmann-Sperlich, Ingo Froboese

**Affiliations:** 1 Institute of Sport Science, Julius-Maximilians-University Würzburg, Würzburg, Germany; 2 Institute of Health Promotion and Clinical Movement Science, German Sport University Cologne, Cologne, Germany; University Heart Center Freiburg, Germany

## Abstract

**Background:**

This study aimed 1) to provide data estimates concerning overall moderate- and vigorous-intensity physical activity (MVPA) as well as MVPA during work, transport and leisure in Germany and 2) to investigate MVPA and possible associations with socio-demographic correlates.

**Methods:**

A cross-sectional telephone survey interviewed 2248 representative participants in the age of 18–65 years (1077 men; 42.4±13.4 years; body mass index: 25.3±4.5kg•m^−2^) regarding their self-reported physical activity across Germany. The Global Physical Activity Questionnaire was applied to investigate MVPA during work, transport and leisure and questions were answered concerning their demographics. MVPA was stratified by gender, age, body mass index, residential setting, educational and income level. To identify socio-demographic correlates of overall MVPA as well as in the domains, we used a series of linear regressions.

**Results:**

52.8% of the sample achieved physical activity recommendations (53.7% men/52.1% women). Overall MVPA was highest in the age group 18–29 years (p<.05), in participants with 10 years of education (p<.05) and in participants with lowest income levels <1.500€ (p<.05). Regression analyses revealed that age, education and income were negatively associated with overall and work MVPA. Residential setting and education was positively correlated with transport MVPA, whereas income level was negatively associated with transport MVPA. Education was the only correlate for leisure MVPA with a positive association.

**Conclusions:**

The present data underlines the importance of a comprehensive view on physical activity engagement according to the different physical activity domains and discloses a need for future physical activity interventions that consider socio-demographic variables, residential setting as well as the physical activity domain in Germany.

## Introduction

There is convincing evidence that physical activity (PA) prevents and treats a wide range of physical and psychological disorders and increases longevity [1]. According to the World Health Organisation's (WHO) estimates, physical inactivity causes approximately 3.2 million world-wide premature deaths per year [2]. Thereby, physical inactivity is one prominent risk factors for several non-communicable diseases, such as coronary heart disease, type 2 diabetes, breast and colon cancer [3]. Therefore the WHO introduced global recommendations for health-related PA prompting adults to at least 150 minutes of moderate-intensity aerobic PA or 75 minutes of vigorous-intensity aerobic PA or an equivalent combination of moderate- and vigorous-intensity PA (MVPA) throughout the week [4]. Accumulation of health-related PA can be obtained thereby in short but multiple bouts of at least 10 min.

Furthermore, the global neglect of health-related PA led to international and national initiatives to promote PA, as evidenced in the recent global call for action within the Toronto Charter for PA [5]. In Germany these initiatives stimulated the development of a national policy action plan on the prevention of malnutrition, inactivity, obesity and connected diseases in 2008. The aim of this plan is to perform audience related actions for every age and to pay special attention to the inactive population [6].

The German National Health Survey introduced recent PA data, indicating that 15.5% of the women and 25.4% of the men are physically active for at least 2.5 hours a week as a proxy for achieving global PA recommendations [7]. The administered questions within the survey consists of: “On how many days of a usual week are you physically active, so that you begin to sweat or you be short of breath?”, while a second question asks the active persons “And how much time do you spend on a typical day with physical activities that let you begin to sweat or be short of breath?”. Answer categories are then “<10 minutes”, “10 to <30 minutes”, “30 to <60 minutes” and “>60 minutes” [7]. Accordingly, a matching with WHO PA guidelines or a comparison with international PA surveillance data is critical because 1) there is no clear differentiation between moderate and vigorous-intensity PA, 2) there is no continuous time-variable because of time categories and 3) there is no consideration of PA of at least 10 minute duration as authors themselves state [7]. Furthermore, the data does not enable a differentiation between different PA domains (4), such as work, transport and leisure which are internationally most common [8], [9]. These information mentioned above however, would give more profound insights where PA de facto appears and opens up for a more distinct and specific PA promotion, which could be especially useful for national policy action by identifying target domains and target groups.

Therefore, the aim of this study was 1) to provide estimates on the prevalence of achieving global PA guidelines within the German population, 2) to provide estimates of MVPA data for the PA domains work, transport and leisure and 3) to investigate possible socio-demographic associations with overall MVPA and MVPA in the domains work, transport and leisure.

## Methods

### Study design and ethical approval

A nationwide cross-sectional questionnaire-based study on health behaviours including questions about self-reported PA was conducted in Germany. The service research centre ‘Growths from Knowledge’ (GfK) in Nürnberg collected data representative for the distribution of the German population between March and April 2012 as part of a computer-assisted telephone interview (CATI). The selected professional interviewers were trained in administering the computer-assisted standardized questionnaire. Due to the telephone survey all participants gave oral informed consent prior to the interview to participate in the study and all study procedures were approved by the Ethics Committee of the German Sport University in Cologne.

### Sample

The sample of 3032 representative residents within the 16 German federal states (1475 men, 1557 women), older than 18 years was chosen from the “ADM pool for telephone samples” (ADM =  **A**rbeitskreis der **d**eutschen **M**arkt- und Sozialforschungsinstitute – study group of German market and social research institutions) [10]. The ADM-pool is the national sample based on all possible telephone-numbers, which forms the selection foundation to create a population sample for the Federal Republic of Germany. The sample was weighted to the German population (year 2011) by age, gender, federal state, residential density and household size according to the data from the National Federal Statistical Office. Only data from adults 18–65 years old were included in the analyses. Finally, the sample size consisted of n = 2248 participants, including 1077 men and 1171 women aged 42.4±13.4 years with a body mass index (BMI) of 25.3±4.5 kg•m^−2^. The participants' socio-demographic characteristics are presented in Table 1.

**Table 1:** Sample characteristics stratified by gender.

	Men	Women	All
N (%)	1077 (47.9)	1171 (52.1)	2248
Age (years)			
18–29 (%)	288 (26.8)	217 (18.5)	505 (22.5)
30–45 (%)	349 (32.4)	421 (35.9)	769 (34.2)
46–65 (%)	440 (40.9)	534 (45.6)	974 (43.3)
Body Mass Index (kg•m^−2^) x±s	25.9±4.3	24.6±4.6	25.3±4.5
Residential setting in inhabitants			
<5.000	178 (16.6)	187 (16.0)	365 (16.3)
5.000–20.000	271 (25.2)	357 (30.5)	628 (28.0)
20.000–100.000	298 (27.6)	309 (26.4)	606 (27.0)
100.000–500.000	146 (13.5)	160 (13.7)	306 (13.6)
>500.000	184 (17.1)	159 (13.5)	342 (15.2)
Education level	*n = 1071*	*n = 1162*	*n = 2233*
No graduation	12 (1.1)	17 (1.5)	29 (1.3)
10 years	167 (15.5)	149 (12.7)	316 (14.1)
12 years	344 (31.9)	472 (40.3)	816 (36.3)
13 years	323 (30.0)	332 (28.3)	655 (29.1)
University degree	225 (20.9)	192 (16.4)	417 (18.5)
Income groups household net income/month	*n = 965*	*n = 1034*	*n = 1999*
<1.500€	327 (30.4)	380 (32.5)	707 (31.5)
1.500–2.999€	418 (38.8)	428 (36.5)	845 (37.6)
>3.000€	220 (20.5)	226 (19.3)	447 (19.9)

Data presents the *n* and (percentage) unless stated otherwise.

### Measures

#### Physical Activity

PA was assessed with the Global Physical Activity Questionnaire (GPAQ) [9] which was designed to measure PA in three domains for a typical week: work (paid and unpaid), transport (i.e., walking and cycling to get to and from places), and leisure activities [9]. Within the work and leisure domains, information on frequency and duration of vigorous-intensity as well as moderate-intensity PA were obtained. For the transport domain, information on all walking and cycling activities were included without a differentiation between the intensity. A single question asks for the usual sitting time on a typical day which results are not presented here. A total of 16 questions were asked in the interview. Weekly minutes of moderate and vigorous-intense activity were calculated separately by multiplying the number of days per week by the duration of an average day. Reported minutes per week in each category were multiplied by the metabolic equivalent (MET; MET-minutes•week^−1^), which is commonly used for expressing intensity of PA independently of body weight. Four METs corresponded to the time spent in moderate-intensity activities and eight METs to the time spent in vigorous-intensity activities (GPAQ analyses guide).

Levels of PA were initially classified into low, moderate and high as defined by the GPAQ analysis framework (http://www.who.int/chp/steps/resources/GPAQ_Analysis_Guide.pdf). The criteria for these levels are:

1. High: Reaching any of the following criteria: (a) Vigorous-intensity activity on at least three days achieving a minimum of at least 1.500 MET-min•wk^−1^ or (b) seven or more days of any combination of walking, moderate- or vigorous-intensity activities achieving a minimum of at least 3,000 MET- min•wk^−1^.
2. Moderate: Meeting any of the following criteria: (a) three or more days of vigorous-intensity activity for at least 20 minutes per day or (b) five or more days of moderate-intensity activity or walking of at least 30 minutes per day or (c) five or more days of any combination of walking, moderate- or vigorous-intensity activities achieving a minimum of at least 600 MET- min•wk^−1^.
3. Low: A person not meeting any of the above mentioned criteria.

Based on the World Health Organization's global recommendations on PA for health [4] we further dichotomized MVPA volume into two categories, inactive (<600 MET-minutes•week^-1^MVPA) and active (>600 MET-minutes•week^−1^MVPA). The term active is thereby defined by accumulating the equivalency of 150 minutes (2.5 h/week) or more of moderate-intensity PA per week and achieving PA recommendations [4] not including any frequency requirement for either moderate- or vigorous-intensity activity.

Validity and reliability have been assessed previously in nine different countries. Concurrent validity between the International Physical Activity Questionnaire (IPAQ) and GPAQ showed a moderate to strong positive relationship (range 0.45 to 0.65) and reliability was of moderate to substantial strength (Kappa 0.67 to 0.73; Spearman's rho 0.67 to 0.81) [11]. Results on criterion validity using pedometer or accelerometer over the duration of 7 days were poor to fair (range 0.06 to 0.35) [11].

#### Socio-demographic Variables

Demographic variables measured self-reported age, gender and body mass index (BMI), calculated using self-reported body weight and body height according to the formula BMI =  body weight (kg) • (body height (m))^−2^. Further socio-demographic variables included the residential density in inhabitants given by the postal code as well as the educational and income level. The educational level was categorized into the following levels based on the German school system: no school graduation, 10 years of education, 12 years of education, 13 years of education and first university degree or higher. Household net income per month was assessed in nine categories and summarized in 3 groups: low income (<1.500€), middle income (1.500€–2.999€), and high income (€>3.000€).

### Data Analysis

Descriptive statistics of percentages were used to determine the prevalence rates for active participants, overall and by socio-demographic variables. Data on overall MVPA as well as for MVPA in the domains work, transport and leisure were analysed using nonparametric analyses for non-normally distributed data, so that medians and quartiles were calculated. The Mann-Whitney test was used when comparing two independent samples (gender) and the Kruskal Wallis test was performed when considering more than two independent samples (age, BMI, residential setting, educational level, income level). Multiple linear regression analyses were executed to investigate associations of socio-demographic correlates and the dependent variables of overall MVPA, work MVPA, transport MVPA and leisure MVPA. We chose the forced entry method to explore the associations. Socio-demographic variables included age (continuous variable), BMI (continuous variable), residential setting (five categories), education (five categories) and income level (three categories). Statistical significance was set at a level of.05 and all analyses were performed using PASW Statistics 20 for Windows.

## Results

### Prevalence of GPAQ-categories and of active participants achieving WHO-PA recommendations

Based on the answers in the interview, 52.8% of the sample achieved PA recommendations (low: 21.7%; moderate: 24.6%; high: 53.3%) and were considered to be active with 53.7% active men and 52.1% active women. Further GPAQ classifications are illustrated in Table 2. The percentage of active participants was highest in the age group 18–29 years with 57.0% compared to 51.1% in the age group 30–45 years and 52.1% in the age group 46–65 years. In the BMI category of 25.0–29.9 kg•m^−2^ the proportion of active participants was highest (54.1%) compared to the other BMI categories. In addition, the proportion of active participants in residential setting with 100.000–500.000 inhabitants was highest (55.8%) compared to the other categories of inhabitants. Concerning education the highest proportion of active participants were found with 10 years of education (61.3%) and lowest proportion in participants with university degree (39.1%), whereas the highest proportion concerning income levels was revealed in the category <1.500€ with 61.0% and lowest proportion of active participants in >3.000€ with 42.7% (Table 2).

**Table 2:** Prevalence of “Global Physical Activity Questionnaire” categories among the 18–65 year-old sample as well as the percentage of active participants meeting WHO guidelines.

	N	Low active	Moderate active	High active	Active (%)
Total sample	2239	21.7	24.6	53.3	52.8
Gender					
Men	1072	20.9	23.7	54.9	53.7
Women	1167	22.4	25.3	51.8	52.1
Age (years)					
18–29	504	15.0	21.9	62.9	57.0
30–45	767	23.3	25.5	50.8	51.1
46–65	968	23.9	25.2	50.3	52.1
Body Mass Index (kg•m^−2^					
<18.5	33	23.6	33.8	41.9	40.5
18.5–24.9	1236	20.8	25.0	53.9	52.9
25.0–29.9	665	22.1	23.4	54.3	54.1
>30	302	24.6	23.5	50.6	51.6
Residential setting in inhabitants					
<5.000	363	27.5	20.8	51.2	53.2
5.000–20.000	627	24.3	23.4	52.0	50.9
20.000–100.000	604	20.2	22.5	57.0	53.9
100.000–500.000	306	15.0	21.7	57.9	55.8
>500.000	338	19.5	32.0	47.3	51.3
Education level					
No graduation	29	15.2	21.5	62.2	55.9
10 years	313	24.3	18.6	56.3	61.3
12 years	814	22.2	20.7	56.9	58.8
13 years	654	20.0	27.6	52.2	49.9
University degree	414	18.8	40.1	41.1	39.1
Income groups household net income/month					
<1.500€	705	16.3	23.4	60.0	61.0
1.500–2.999€	842	22.7	22.9	54.0	53.1
>3.000€	446	25.6	28.2	46.0	42.7

Sample stratified by gender, age, BMI, residential setting, education and income level.

Data presents the percentage.

### Overall MVPA and MVPA in work, transport and leisure domain

Table 3 summarizes the medians and quartiles of MVPA MET-minutes in overall MVPA and the different domains accounted by gender, age, BMI, residential setting, education and income level.

**Table 3:** MVPA MET-minutes•week^−1^ in overall MVPA and in the domains work, transport and leisure. Sample is stratified by gender, age, BMI, residential setting, education and income.

	Overall MVPA (MET-minutes•week^−1^)	Work MVPA (MET-minutes•week^−1^)	Transport MVPA (MET-minutes•week^−1^)	Leisure MVPA (MET-minutes•week^−1^)
Total sample	630	120	60	180
	(270–1620)	(0–1050)	(0–210)	(30–360)
Gender				
Men	660	60	60	180
	(270–1800)	(0–1200)	(0–240)	(0–360)
Women	630	120	60	150
	(270–1500)	(0–900)	(0–210)	(45–300)
Age (years)				
18–29	720 a ^,^ b	120	90 a ^,^ b	240 a ^,^ b
	(330–2145)	(0–1440)	(0–300)	(80–420)
30–45	600	90	60	120
	(240–1500)	(0–1080)	(0–210)	(6–270)
46–65	601	120	60	150
	(270–1470)	(0–840)	(0–210)	(0–358)
Body Mass Index (kg•m^−2^)				
<18.5	471	180	60 c	150
	(289–1466)	(0–690)	(0–138)	(48–279)
18.5–24.9	630	6^d^^,^ e	89^d^^,^ e	180 d ^,^ e
	(270–1500)	(0–840)	(0–270)	(60–360)
25.0–29.9	645	180	60	135
	(270–1786)	(0–1259)	(0–210)	(0–300)
>30	660	240	60	120
	(240–1860)	(0–1479)	(0–180)	(0–300)
Residential setting in inhabitants				
<5.000	663	144 i	0 f ^,^ g ^,^ h ^,^ i	180
	(265–1554)	(0–1080)	(0–146)	(60–360)
5.000–20.000	600	120 l	40 j ^,^ k ^,^ l	162
	(240–1470)	(0–948)	(0–188)	(59–270)
20.000–100.000	714	164 n	80 m ^,^ n	179
	(300–1800)	(0–1200)	(0–240)	(0–360)
100.000–500.000	690	60	140	180
	(315–1509)	(0–872)	(0–300)	(59–416)
>500.000	600	0	120	150
	(240–1489)	(0–840)	(0–300)	(0–360)
Education level				
No graduation	882 r	315 q ^,^ r	97 o ^,^ p ^,^ q ^,^ r	138
	(405–4740)	(0–3360)	(0–600)	(0–260)
10 years	912 t ^,^ u	360 t ^,^ u	60	105 s ^,^ t ^,^ u
	(300–2160)	(0–1520)	(0–270)	(0–274)
12 years	780 v ^,^ w	360 v ^,^ w	60 v	160 v
	(300–2160)	(0–1520)	(0–270)	(0–300)
13 years	589 x	0 x	90	180
	(270–1440)	(0–720)	(0–240)	(60–360)
University degree	450	0	60	180
	(210–860)	(0–240)	(0–200)	(90–360)
Income groups household net income/month				
<1.500€	900 y ^,^ z	240 y ^,^ z	90 y ^,^ z	140
	(360–2160)	(0–1500)	(0–270)	(0–360)
1.500–2.999€	630 aa	120 aa	60 aa	180
	(270–1666)	(0–1035)	(0–210)	(27–315)
>3.000€	480	0	50	180
	(185–1149)	(0–480)	(0–180)	(90–360)

Data presents the median and the (quartiles). Statistical difference was set by p<.05.

a Age group 18–29 years differs significantly from age group 30–45 years.

b Age group 18–29 years differs significantly from age group 46–65 years.

c Participants with a BMI of <18.5-kg•m^−2^ differ significantly from participants with BMI of 18.5–24.9 kg•m^−2^.

d Participants with a BMI of 18.5–24.9 kg•m^−2^ differ significantly from participants with BMI of 25.0–29.9 kg•m^−2^.

e Participants with a BMI of 18.5–24.9 kg•m^−2^ differ significantly from participants with BMI of>30 kg•m^−2^.

f Participants living in areas with <5.000 inhabitants differ from participants living in areas 5.00–20.000 inhabitants.

g Participants living in areas with <5.000 inhabitants differ from participants living in areas 20.00–100.000 inhabitants.

h Participants living in areas with <5.000 inhabitants differ from participants living in areas 100.00–500.000 inhabitants.

i Participants living in areas with <5.000 inhabitants differ from participants living in areas>500.000 inhabitants.

j Participants living in areas with 5.000–20.000 inhabitants differ from participants living in areas 20.00–100.000 inhabitants.

k Participants living in areas with 5.000–20.000 inhabitants differ from participants living in areas 100.000–500.000 inhabitants.

l Participants living in areas with 5.000–20.000 inhabitants differ from participants living in areas>500.000 inhabitants.

m People living in areas with 20.000–100.000 inhabitants differ from participants living in areas 100.00–500.000 inhabitants

n Participants living in areas with 20.000–100.000 inhabitants differ from participants living in areas>500.000 inhabitants.

o Participants with no graduation differ from participants with 10 years of education.

p Participants with no graduation differ from participants with 12 years of education.

q Participants with no graduation differ from participants with 13 years of education.

r Participants with no graduation differ from participants with university degree.

s Participants with 10 years of education differ from participants with 12 years of education.

t Participants with 10 years of education differ from participants with 13 years of education.

u Participants with 10 years of education differ from participants with university degree.

v Participants with 12 years of education differ from participants with 13 years of education.

w Participants with 12 years of education differ from participants with university degree.

x Participants with 13 years of education differ from participants with university degree.

y Participants with <1.500 € household net income/month differ from participants 1.500–2.999€ household net income/month.

z Participants with <1.500 € household net income/month differ from participants>3.000€ household net income/month.

aa Participants with 1.500–2.999 € household net income/month differ from participants>3.000€ household net income/month.

There were no differences in MVPA concerning gender. Participants in the age group 18–29 years showed higher overall MVPA as well as higher transport and leisure MVPA than the older age groups. Participants with a BMI in the range of 18.5–24.9 kg•m^−2^ had lower work MVPA and higher transport and leisure MVPA than the BMI groups 25.0–29.9 kg•m^−2^ and >30.0 kg•m^−2^. The main result for the residential setting is that by increasing number of inhabitants transport MVPA increases as well. Concerning the level of education it is shown that participants with 10 or 12 years of education have higher overall MVPA as well as work MVPA, but lower leisure MVPA than participants with 13 years of education or university degree. Participants in the lowest income category have higher overall MVPA as well as higher work and transport related MVPA than the higher income levels.

### Results of multiple linear regression analyses

Multiple linear regression analyses showed that 3.5% of the variance (adjusted R^2^) in overall MVPA, 5.1% in work MVPA, 2% in transport MVPA and 1.3% of the variance in leisure MVPA were explained by the variables entered in the model (Table 4). Age, education and income were negatively associated with overall and work MVPA, indicating that increasing age, education and income level leads to lower overall and work MVPA. BMI was positively associated with overall and work MVPA meaning that increasing BMI is associated with increasing overall and work MVPA. Residential setting and education was positively correlated with transport MVPA, showing higher transport MVPA in settings with higher numbers of inhabitants and increasing education. Income level was negatively associated with transport MVPA. The only significant variable for leisure MVPA was education, indicating a higher education level with higher leisure MVPA.

**Table 4:** Results from multiple linear regressions on contribution of socio-demographic correlates on the dependant variable “Overall MVPA”, “Work MVPA”, “Transport MVPA” and “Leisure MVPA”.

	Overall MVPA (n = 1962)			Work MVPA (n = 1972)			Transport MVPA (n = 1993)			Leisure MVPA (n = 1992)		
	B	SE B	β	B	SE B	β	B	SE B	β	B	SE B	β
Gender	-83.0	63.9	-.03	-96.5	54.2	-.04	32.0	20.9	.04	-18.4	16.0	-.03
Age	-7.6	2.5	**-.07** **	-6.7	2.1	**-.07** **	-.6	.8	-.02	-.5	.6	-.02
BMI	14.5	7.3	**.05** *	18.6	6.2	**.07** **	-2.0	2.4	-.02	-2.3	1.8	-.03
Residential setting	27.9	25.0	.03	5.7	21.2	.01	24.5	8.2	**.07** **	-1.6	6.2	-.01
Education level	-66.9	31.0	**-.05** *	-134.8	26.3	**-.12** ***	31.4	10.1	**.07** **	33.0	7.7	**.10** ***
Income level	-256.4	44.6	**-.14** ***	-193.2	37.8	**-.12** ***	-61.4	14.6	**-.10** ***	-4.5	11.2	-.01
	*Adj. R^2^* = .035			*Adj. R^2^* = .051			*Adj. R^2^* = .02			*Adj. R^2^* = .013		

B =  unstandardized beta; SE B =  standard error of beta; β =  standardized beta;

* = p<0.05;

** = p<0.01;

*** = p<0.001.

## Discussion

Previous studies conducted in Germany and monitoring levels of PA examined merely the overall MVPA [7], [12], [13], [14], leisure PA [15] and/or applied questionnaires that do not allow for an international comparison [7], [14], [15]. For the first time however, the present study investigated the prevalence of self-reported MVPA in Germany in different PA domains, i.e. work, transport and leisure using an internationally accepted and comparable questionnaire.

### PA recommendations

Overall, 53.7% men and 52.1% women of the 18–65 year olds were considered as “active” and meeting PA recommendations for health [4], which is explicitly higher than the results of the most recent data from the German National Health Survey [7]. This may be partially explained by the different PA indicators used in the present study, asking for moderate- as well as for vigorous-intense PA, compared to the national surveillance study [7]. Furthermore, in the present study the questionnaire explicitly asked for every PA domain, including work as well as transport, separately, compared to the overall question used in the national surveillance which may lead to a further underestimation.

Compared to the international Eurobarometer 58.2 study from 2002, applying the IPAQ short, where 46.1% of German men and 34.7% of German women achieved the PA guidelines [12], we also have a higher proportion of active men and women in the present study. Differences may be explained through increasing PA levels, which are reported on in some surveillance studies [7], [16] or through the only acceptable association between the IPAQ and the GPAQ assessment tool [11]. Another reason for the rather high results in the present study could be the noticeable high contribution of work (mean: 38.1%) and transport MVPA (mean: 24.8%) to overall MVPA, which has not been assessed specifically in the previous mentioned surveillance studies [7], [12]. In respect to these higher rates of MVPA, it has to be questioned whether work-related MVPA, which is often monotone, unidirectional and/or repetitive has the same health contribution as MVPA in the transport and leisure domain. For example as it has been stated before work-related PA seems to be a significant work-related risk factor for back pain [17]. Considering only transport and leisure MVPA for achieving 600 MET-min, then only 22.7% of the overall sample, respectively 23.5% of men and 21.9% of women would achieve PA recommendations. Notably, these results are much more in the range of the national surveillance study [7]. Here future research is warranted, investigating whether work-related MVPA has the same health contribution as MVPA in the transport and leisure domain.

Referring to the IPS study of PA prevalence [18] and the recent study on global PA levels [19] stating that worldwide 31.1% are considered inactive, Germany with the present 21.7% inactive participants is in the lower range of inactivity, which however, still outlines the need for PA promotion.

### Correlates of overall MVPA and MVPA in the domains work, transport and leisure

One aim of the present study was to explore overall MVPA as well as MVPA in the domains work, transport and leisure in respect to different socio-demographic correlates. Multivariate models explained more of the variance in work MVPA (R^2^ = 5.1%) compared to overall MVPA (R^2^ = 3.5%), transport MVPA (R^2^ = 2%) and leisure MVPA (R^2^ = 1.3%). The results showed that a large part of the model variance remains unexplained by the included correlates which is mainly due to not including psychological, behavioural, social or environmental correlates which play an important role in explaining adults PA behaviour [20]. However, from a public health perspective, the low variance is still of significance for developing interventions to special population target groups in Germany.

We neither found differences nor significant association between gender and MVPA, which is in contrast to [21] as well as the results of recent German data, which demonstrates that men achieve PA recommendations more often than women [7]. However our results are in line with the inconclusive result on gender and PA of the systematic review of [22], which shows that there is no definitive direction for PA in gender. Our results indicate future PA promotion for men and women in the same degree.

Regarding age, our results support previous findings that younger age groups are more involved in overall MVPA [13], [20], [21], transport MVPA [23] and leisure-time MVPA [7], [20]. However, we only found a negative association of age with total MVPA and work MVPA, which points out that age is only an ostensible influencing factor on transport and leisure MVPA and not a crucial correlate in the present study. These contradicting findings concerning leisure MVPA could be due to the age-cut off of ≤65 years in the present study compared to [7], [20]. Yet, our descriptive data reveal that at least 25% of the sample in the age between 46–65 years is not participating in any transport or leisure MVPA, which is crucial in regard of overall health. As [24] shows PA program offers in the age <60 years are only sporadic in the field of primary prevention in Germany and needs a stronger focus, as well as the active travel promotion.

With respect to BMI, we found higher transport and higher leisure MVPA in normal-mass participants compared to overweight and obese participants. These finding are supported by former studies [21], [25], [26], [27]. However, we neither detected a significant negative association between transport MVPA and BMI nor between leisure MVPA and BMI, but a positive association between BMI and overall MVPA as well as work MVPA. This surprising positive association on overall MVPA is in contrast to [21] and could be partially explained by the high mean proportion of work MVPA of overall MVPA in overweight (42.3%) and obese (46.6%) participants. A reason for the positive association in BMI with work MVPA could be due to a physically inactive behaviour after work for the rest of the day [22] and maybe through coexistent negative behaviour such as high caloric intake in physically hard working staff. Nevertheless, this finding reveals the need for specific health promotion in physically hard working populations, or the so-called blue-collar workers.

The present study acknowledged the finding of others [28] that people living in rural surrounding have higher work MVPA than people living in urban setting. The finding that transport MVPA was positively associated with increasing number of inhabitants was contrary to the findings of Poland [29], where active commuting was not very common all around the country. However, present data resembles the results of Belgium and Switzerland [30], [31] and may be explained through environmental correlates that are known to support transport MVPA such as residential density, street connectivity, land use mix etc. [32], which are more frequent in urban settings. Concerning leisure PA, we did not find any tendency for residential setting and concerning this topic in the literature there are mixed findings [28], [31], [33], which may be due to different situations in each country. However, our results indicate a specific need for active travel promotion especially in the more rural setting.

The results concerning the education show two opposing influences on MVPA. Finding a positive association of leisure MVPA with higher education levels was expected and is in line with several study outcomes before [21], [34], [35], [36], [37] and underlines the importance of implementing PA promotion in population groups with low education level. Additionally, we found an independent positive association between transport MVPA and education. Overall there are mixed findings in the literature [37], showing negative, and positive as well as null associations. It can be assumed that external factors such as the neighbourhood environment, access to public transport, transportation infrastructure might be especially important for transport MVPA, so that more research especially for Germany here is recommended. In contrast, present results indicate that with lower education levels higher work MVPA is apparent, which is in accordance with previous findings [35], [37], [38]. Noteworthy, in the present study the strong negative work MVPA association masks the positive associations of transport and leisure, which leads to a negative association for overall MVPA and shows what a substantial role work MVPA still has in especially low education groups. This explains also the finding of recent German results [34] that lower educated population groups achieve the proxy of PA recommendations more often than higher educated population groups. In respect to the questionable health effect of work MVPA PA promotion in Germany must be directed towards the domains transport and leisure especially in lower educated population groups.

The PA pattern for income levels were in overall MVPA and work MVPA similar to education, but showed a different independent, namely negative association for transport MVPA and income level. This may be explained through the, especially in Germany common “status symbol” of driving a car instead of using public transport, taking the bike or walk. Here, it seems reasonable to promote active transportation especially also in populations with higher income levels in Germany.

The analyses of PA in different domains are among the strengths of the study and are unique for Germany. Nevertheless, some limitations of the current study should be noted. The used questionnaire was especially realized for developing countries, [9] where PA patterns may differ from developed countries because work-, domestic-, and transport-related activities may contribute more to overall PA than leisure-time or recreational activity. Most previous monitoring studies which included European countries used the IPAQ [12], [18], [39], which reduces the compatibility between the studies. The underlying reason for the choice of questionnaire was due to the objective to investigate overall PA as well as PA in the different domains, but with fewer questions than the IPAQ long. However, research comparing IPAQ with GPAQ results in developed countries is warranted.

A crucial element in the present study is the overall length of the survey which included other health behaviors as well. This could lead to bias in the answering of PA questions and needs to be considered. Moreover, using a questionnaire to assess PA may result in a misclassification of PA behaviour, due to the use of self-reported measures which can lead to an over-estimation of PA [40]. Therefore, studies using objective measures such as accelerometers are eligible. Furthermore, it is important to note that our results are limited to PA behaviour in spring season, and we would expect to see seasonal variations in PA between the other seasons of the year [41].

## Conclusion

To our knowledge, this is the first study reporting on MVPA concerning the domains of work, transport and leisure of a representative German population with an internationally accepted assessment tool. The data revealed differences in overall MVPA between age groups, education and income levels and acknowledged major discrepancy regarding the PA patterns of the participants in the different PA domains. The results disclosed primary action in promoting active transport especially for older adults, in rural settings, and in people with higher income and reveals the need for leisure PA promotion especially in older adults, in overweight adults, and in population groups with lower education level. The study further underlines the need for a comprehensive and ongoing view on PA behaviour in the different domains and explains the need for further policy action, i.e. in concise targets for domain-specific PA in Germany which considers socio-demographic variables as well as environmental surroundings.
